# Factors Associated with COVID-19 Infection Related Multisystem Inflammatory Syndrome in Children: A Multicenter Matched Case-Control Study

**DOI:** 10.3390/children12060678

**Published:** 2025-05-24

**Authors:** Buddhaporn Prasertsakul, Phanthila Sitthikarnkha, Chetta Ngamjarus, Chaniya Jakeaw, Sumitr Sutra

**Affiliations:** 1 Department of Pediatrics, Chum Phae Hospital, Khon Kaen 40130, Thailand; b.prasertsakul@gmail.com; 2 The Office of Disease Prevention and Control Region 7, Department of Disease Control, Ministry of Public Health, Khon Kaen 40000, Thailand; 3 Department of Pediatrics, Faculty of Medicine, Khon Kaen University, 123 Mittraphap Road, Muang, Khon Kaen 40002, Thailand; sumitr@kku.ac.th; 4 Department of Epidemiology and Biostatistics, Faculty of Public Health, Khon Kaen University, Khon Kaen 40002, Thailand; nchett@kku.ac.th; 5 Department of Pediatrics, Khon Kaen Hospital, Khon Kaen 40000, Thailand; ch.jakaew@cpird.in.th

**Keywords:** multisystem inflammatory syndrome, children, MIS-C, COVID-19, coronavirus 2019

## Abstract

Background/Objectives: After pandemic of COVID-19, there were increased the incidence of Multisystem Inflammatory Syndrome in Children (MIS-C), as reported by the Centers for Disease Control and Prevention (CDC). However, it remains unclear which specific factors link MIS-C to COVID-19 following infection. This study aims to investigate the factors associated with MIS-C in children infected with COVID-19. Methods: A multicenter-matched case-control study was conducted across Chum Phae, Khon Kaen, and Srinagarind Hospitals, Thailand. We included patients under 21 years old from those hospitals from January 2021 to February 2024. The cases were patients diagnosed with MIS-C, while the controls had a history of COVID-19 infection but had not been diagnosed with MIS-C at least 3 months post-infection. The matching criteria for cases and controls, in a 1:2 ratio, included gender and age. The association between various factors and MIS-C was examined using conditional logistic regression. Results: A total of 34 MIS-C cases were matched with 68 controls. We found that antiviral therapy administered during COVID-19 infection was linked to a reduced risk of MIS-C development, with an adjusted odds ratio of 0.06 (95% CI: 0.02–0.20). However, this study found no association between COVID-19 vaccination and nutritional status in the development of MIS-C. Conclusions: The administration of antiviral treatment during COVID-19 infection was associated with a diminished incidence of MIS-C.

## 1. Introduction

Severe Acute Respiratory Syndrome Coronavirus-2 (SARS-CoV-2), or COVID-19, has been identified since December 2019. It causes a highly contagious infection that quickly spreads globally. The World Health Organization (WHO) declared a global pandemic in March 2020 [1]. Subsequently, in April 2020, cases of children presenting with symptoms similar to Kawasaki disease were reported [2]. The National Health Service (NHS) of the United Kingdom warned that these symptoms were associated with COVID-19 in children. Later, countries in the European Union and the United States reported a syndrome characterized by multisystem inflammatory symptoms in pediatric patients [3], which could be severe enough to cause hypotension requiring vasopressor support and necessitate mechanical ventilation due to cardiovascular involvement rather than respiratory failure [4]. The WHO defined this condition as a temporary multisystem inflammatory syndrome in children and adolescents related to COVID-19. The Centers for Disease Control and Prevention (CDC) in the United States termed it Multisystem Inflammatory Syndrome in Children (MIS-C). The Royal College of Pediatrics and Child Health (RCPCH) in the United Kingdom referred to it as Pediatric Inflammatory Multisystem Syndrome Temporally Associated with SARS-CoV-2 (PIMS-TS) [4].

Current studies indicate that MIS-C typically occurs after COVID-19 infection rather than during the active infection. The clinical manifestations of MIS-C exhibited notable similarities to various other conditions, particularly Kawasaki disease and toxic shock syndrome [5], which can lead to delays and confusion in diagnosis during the initial stages. Following an overwhelming inflammatory response, the clinical manifestations and indicators of multiorgan dysfunction will promptly become evident. Timely diagnosis of MIS-C is essential for healthcare professionals; equally important is the identification of factors linked to the onset of MIS-C subsequent to COVID-19 infection. Understanding these factors can aid in the development of targeted interventions and improve patient outcomes. There was a study of MIS-C occurring after COVID-19 vaccination in children, with varying efficacy depending on the type of vaccine [6]. However, some studies found that vaccination against SARS-CoV-2 infection played an important role in reducing the risk of developing MIS-C [7,8].

The data on associated factors for MIS-C development among children were scarce. Understanding the factors could enable better surveillance, timely diagnosis, and treatment of this syndrome. Additionally, early intervention could potentially reduce mortality rates associated with delayed diagnosis. Thus, the primary objective of this study was to explore the factors associated with MIS-C in children infected with COVID-19.

## 2. Materials and Methods

### 2.1. Study Design and Participants

A multicenter matched case-control study was conducted involving individuals under 21 years of age who had been hospitalized at Chum Phae Hospital, Khon Kaen Hospital, and Srinagarind Hospital from 1 January 2021 to 29 February 2024. These hospitals have classified the hospital level as secondary, tertiary, and super-tertiary, respectively. The cases in this study included patients diagnosed with MIS-C, while the controls were individuals with a history of COVID-19 infection who had not been diagnosed with MIS-C at least three months post-infection. The matching criteria for cases and controls were established in a 1:2 ratio, considering factors such as gender and age. Participants who were diagnosed with Kawasaki disease, staphylococcal scalded skin syndrome, and toxic shock syndrome were systematically excluded from the study. Excluding these conditions will minimize confounding variables and ensure that the results were solely attributable to the primary factors being studied.

A sample size was calculated for a matched case-control study utilizing the appropriate formula [9]. From the research by Zambrano et al. [10], we determined the proportion of exposure to be 0.049, with an odds ratio (OR) of 8. The significance level for Type I error was established at 0.05, while the probability of Type II error was determined to be 0.2. Consequently, we arrived at a sample size of 34 cases and 68 controls.

### 2.2. Data Collection

Cases were defined as patients diagnosed with MIS-C based on the consensus of multidisciplinary pediatricians and the diagnostic criteria established by the Centers for Disease Control and Prevention (CDC), which includes the following criteria: (1) Body temperature above 38 °C persisting for over 24 h or subjective fever lasting longer than 24 h, (2) laboratory findings of inflammation, demonstrated by increased levels of C-reactive protein (CRP), erythrocyte sedimentation rate (ESR), fibrinogen, procalcitonin, D-dimer, ferritin, lactate dehydrogenase (LDH), interleukin-6 (IL-6), and neutrophils, coupled with reduced levels of lymphocytes and albumin, (3) Multisystem involvement is characterized by clinically severe illness that necessitates hospitalization and affects at least two organ systems, which may include cardiac, renal, respiratory, hematologic, gastrointestinal, or dermatologic systems, (4) symptoms sufficiency severe enough to require hospitalization, and 5) Evidence supporting a prior COVID-19 infection within four weeks preceding the onset of symptoms can be established by any one of the following: a positive result for SARS-CoV-2 via reverse transcription polymerase chain reaction (RT-PCR), the presence of antibodies against SARS-CoV-2, an antigen test, or documented exposure to COVID-19 [11].

The participants in the control group were hospitalized patients with a history of SARS-CoV-2 infection confirmed by positive antigen test kit, or RT-PCR but did not develop MIS-C within three months post-infection. Controls were selected and matched to cases in a 1:2 ratio based on age (within one year) and gender. The management of pediatric patients who were hospitalized due to COVID-19 was performed in adherence to the Thai National Treatment Guidelines for COVID-19 across all hospitals [12].

We collected extensive data in both the case and control groups on various significant factors, including the patients’ age at the time of COVID-19 infection, gender, nutritional status, history of COVID-19 vaccination, clinical symptoms noted at the time of admission, laboratory findings, and treatment strategies applied during COVID-19 infection.

### 2.3. Statistical Analyses

Categorical variables were presented as frequencies and percentages, and differences between the two groups were compared using Pearson’s Chi-squared test or Fisher’s exact test. Median and interquartile ranges (IQR) were used to describe the outcomes of continuous variables, and the Wilcoxon rank sum test was used to calculate differences in medians of certain continuous variables. The associated factors of MIS-C was investigated using both simple and multiple conditional regression analyses, with results presented as crude and adjusted odds ratios (ORs) and 95% confidence intervals (95% CIs). The multivariable conditional logistic regression model incorporated variables including nutritional status, vaccination history, and the administration of antiviral medications. R Statistical Software Version 4.4.1 (R Core Team 2024) was used to analyze all data in this study.

## 3. Results

### 3.1. Demographic Data

A total of 34 patients diagnosed with MIS-C were enrolled as cases in this study. Among these patients, 16 (47.1%) had no prior documented cases of SARS-CoV-2 infection. However, seven of these individuals tested positive for SARS-CoV-2 via RT-PCR, indicated by low cycle threshold values, suggesting a prior COVID-19 infection. Additionally, four patients exhibited the presence of anti-nucleocapsid (anti-N) antibodies against SARS-CoV-2, indicating past COVID-19 infection rather than vaccination-derived immunity. Furthermore, five patients reported documented exposure to COVID-19 within four weeks prior to the onset of their clinical symptoms. All these MIS-C patients were classified as cases. A total of 68 hospitalized patients with a history of SARS-CoV-2 infection were recruited in the control group. Case and control patients were well-matched in gender and age.

Malnutrition was found to be more common among participants in the case group compared to those in the control group. Notably, the control group had two individuals with a history of asthma, whereas no participants in the case group reported such a history. The vaccination history for the case group was recorded before the diagnosis of MIS-C. In contrast, in the control group, vaccination history was documented both before and three months after SARS-CoV-2 infection. The individuals participating in this study exclusively utilized the mRNA-based vaccine developed by the Pfizer-BioNTech COVID-19 Vaccine^®^ (Kalamazoo, MI, USA). The case and control groups consisted of a small number of participants who had received at least two doses of the vaccine, with five individuals (14.7%) in the case group and five individuals (7.4%) in the control group. There is no significant difference in duration after the first, second, and last doses of the vaccine and the occurrence of COVID-19 infection (Table 1).

### 3.2. Clinical Characteristics

Clinical symptoms during the SARS-CoV-2 infection period in the control group (68 controls) showed greater systemic involvement compared to the case group (18 cases) as follows: upper respiratory tract symptoms were present in 64.2% versus 33.3%, lower respiratory tract symptoms in 60.3% versus 27.8%, gastrointestinal symptoms in 20.6% versus 11.1%, and neurological symptoms in 22.1% versus 0%. In terms of the severity of SARS-CoV-2 infection, most cases in both groups exhibited mild symptoms. Complications, including respiratory failure, acute laryngotracheitis, and febrile seizures, were noted in approximately 5% of both groups. In the case group, one patient experienced respiratory failure requiring mechanical ventilation, while in the control group, three patients had febrile seizures, and one patient had acute laryngotracheitis (Table 2).

**Figure 1:**
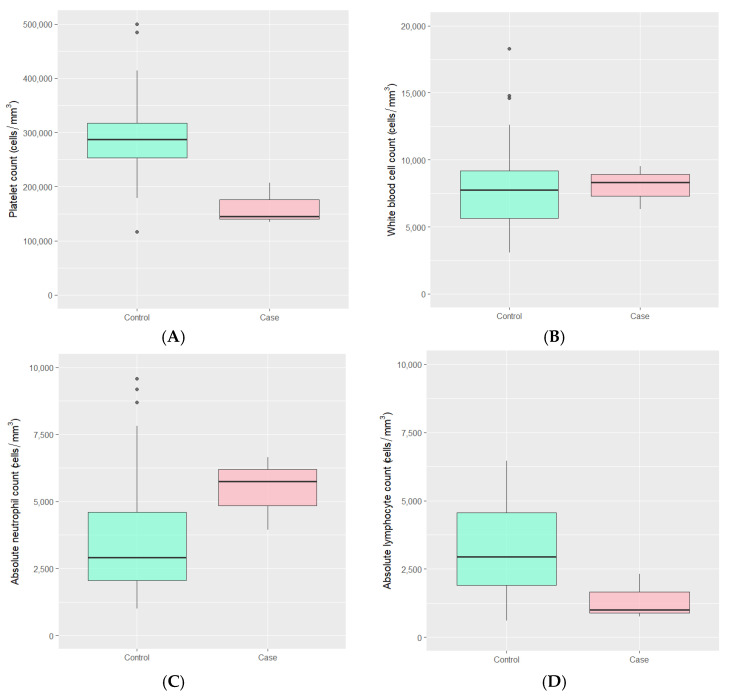
The distributions of (**A**) platelet count, (**B**) white blood cell count, (**C**) absolute neutrophil count, and (**D**) absolute lymphocyte count during COVID-19 infection among MIS-C case children and hospitalized control children with COVID-19 infection.

Laboratory findings during the SARS-CoV-2 infection period in both groups were typically within normal ranges. However, the median absolute lymphocyte count was lower in the MIS-C group, with values of 996 (IQR 882, 1660) compared to 2949 (IQR 1907, 4551) cells/mm^3^ in the control group (*p*-value 0.041). Additionally, the median platelet count in the MIS-C group was lower than that in the control group, with values of 145,000 (IQR 140,000, 176,000) versus 287,000 (IQR 253,000, 317,500) cells/mm^3^ (*p*-value 0.011). There were no statistically significant differences observed in the total white blood cell count and absolute neutrophil count between the two groups (Figure 1 and Table 2).

In the case group, only 8 individuals (44.4%) received antiviral drugs during their SARS-CoV-2 infection, while 18 had a history of infection. One individual received a 10-day course of remdesivir due to respiratory failure. In the control group, 53 individuals (77.9%) received antiviral drugs, all of whom were treated with favipiravir (Table 2).

### 3.3. Factors Associated with MIS-C

Due to the significant amount of missing clinical and laboratory data during the infection period, these data were not analyzed for the factors of interest. Children who receive antiviral therapy, including favipiravir and remdesivir, exhibit a reduction in the risk of MIS-C, with an adjusted OR of 0.06 (95% CI: 0.02–0.20). The nutritional status, encompassing malnutrition, overweight, and obesity, demonstrates adjusted ORs of 0.79 (95% CI: 0.23–2.70), 0.93 (95% CI: 0.22–3.96), and 0.44 (95% CI: 0.07–2.92), respectively. Furthermore, children who received one dose or at least two doses of the SARS-CoV-2 vaccine have adjusted ORs of 0.28 (95% CI: 0.06–1.27) and 1.83 (95% CI: 0.34–9.89), respectively (Table 3).

## 4. Discussion

This multicenter study analyzed the clinical features of children with MIS-C against control cases of COVID-19 in Thailand. Remarkably, only half of the children with MIS-C in our research exhibited the typical clinical signs or a confirmed COVID-19 diagnosis before developing MIS-C. In contrast, a positive RT-PCR result for SARS-CoV-2 or the detection of antibodies against SARS-CoV-2 was required to validate the MIS-C diagnosis. The comprehensive investigation focused on augmenting the precision of evidence related to COVID-19 infections revealed the presence of COVID-19 antibodies in four study participants. These antibodies act as markers for exposure to COVID-19, even in the absence of clinical manifestations and without definitive confirmation of the virus. This underscores the importance of SARS-CoV-2 antibodies for accurate diagnosis. Moreover, these antibodies were found to be detectable for at least 90 days following the onset of the infection, as noted in prior studies [7]. To effectively differentiate between antibody responses associated with COVID-19 infection, an increase in anti-nucleocapsid antibodies should be employed to delineate COVID-19 infection from post-vaccination responses, which typically demonstrate only anti-spike antibodies [13]. This understanding holds substantial promise for optimizing the timing of diagnoses and enabling early intervention by enhancing comprehension of immune response patterns in relation to COVID-19 exposure.

In terms of demographic data, our findings aligned with earlier studies, showing no significant differences in gender distribution among cases [5,14,15]. Children diagnosed with MIS-C in our study had the median age of 6.7 years, which is lower than what has been reported in previous studies [16,17,18]. The differences in age reported across various studies underscore the unique challenges that children encounter in different regions. This variation can be attributed to the timing of data collection, which affects how and when vaccines are administered in different countries. In Thailand, COVID-19 vaccines have been administered to children over 12 years old since 17 August 2021, and to those aged 5 years and older since 10 February 2022 [19]. COVID-19 vaccines play a crucial role in enhancing immunity and reducing the severity of infections. A study conducted on adults found that after receiving two doses of the Pfizer-BioNTech BNT162b2 mRNA vaccine (Kalamazoo, MI, USA), the levels of IgG antibodies surged by over 2000 times at one month after vaccination [20]. Additionally, a study focusing on adolescents aged 12 to 15 demonstrated a 50% increase in immunity against COVID-19 after the second dose, comparable to the 16 to 25 age group [21]. Therefore, a larger proportion of adolescents received vaccinations compared to younger children throughout the duration of this study, resulting in a lower reported median age relative to earlier studies.

In this study, 18 cases (52.9%) reported a history of COVID-19 infection. Previous research has shown a variation in the evidence of COVID-19 infection prior to MIS-C, ranging from 29% to 71% [17,18]. Discrepancies may arise from variations in testing and treatment accessibility across countries, as well as the wide range of COVID-19 symptoms, particularly among children, which can vary from asymptomatic to severe [22]. This variability may result in certain asymptomatic and mild cases not receiving a confirmed diagnosis of COVID-19 infection. In the control group, the most frequently reported clinical symptoms during COVID-19 infection included upper respiratory symptoms, followed by lower respiratory, gastrointestinal, and neurological symptoms, all of which were more prevalent than in the case group. This is likely due to the fact that 47% of the case group had no history of infection, contributing to an underreporting of symptoms.

Regarding factors associated with COVID-19 MIS-C, our findings indicate that antiviral treatment during COVID-19 significantly correlates with a reduction in MIS-C, evidenced by an adjusted OR of 0.06 (95% CI 0.02–0.20). In accordance with the Thai COVID-19 Clinical Practice Guidelines for the diagnosis and treatment of SARS-CoV-2 infection, the antiviral medications administered during the study comprised favipiravir and remdesivir [12]. Children with mild to moderate symptoms who required hospitalization predominantly received favipiravir, which resulted in favorable clinical outcomes and minimal adverse effects, as noted in prior studies [23]. The observed decrease in MIS-C incidence among these children may be attributed to the ability of antivirals like favipiravir and remdesivir to shorten recovery times and modulate the immune response post-infection. A randomized controlled trial involving 1062 hospitalized adults revealed that patients treated with remdesivir had a lower median recovery time of 10 days than those who were administered a placebo, which had an average recovery time of 15 days [24]. Additionally, a systematic review with meta-analysis showed that patients receiving favipiravir had significantly better clinical outcomes compared to the control group after 7 days of hospitalization. However, the viral clearance at 14 days after infection were not statistically significant [25]. Notably, this study is the first to demonstrate a significant connection between antiviral treatment during COVID-19 infection and the occurrence of MIS-C.

The COVID-19 vaccination data analyzed in this study did not demonstrate a statistically significant relationship with the occurrence of MIS-C. However, previous research has indicated that vaccination may contribute to a reduction in the incidence of MIS-C. In earlier studies, it was observed that among MIS-C patients, approximately 10% had received one dose of the COVID-19 vaccine, while around 33% had received two doses. The incidence rate of MIS-C in the previous study was estimated to be approximately 1 case per million vaccinated individuals within the age group of 12 to 20 years [17,26]. The discrepancy in our results may be attributed to the fact that the MIS-C patients in our study were younger than those in earlier studies and may have been recipients of the vaccine later on.

There were several limitations in this study. Firstly, its retrospective nature may result in some gaps in the data collection process. Secondly, the small sample size restricts the possibility of conducting some subgroup analyses. Nonetheless, considering the low prevalence of MIS-C, the calculated sample size for the matched case-control study provided adequate statistical power for the data analysis. Thirdly, some patients diagnosed with MIS-C in this study had a history of COVID-19 exposure rather than confirmation through RT-PCR or antibody testing. Nevertheless, they fulfilled the criteria for MIS-C as outlined by the CDC and WHO. Additionally, details regarding treatment and outcomes were not included in this study. Future research could potentially analyze the effects of remdesivir and favipiravir separately to yield more specific insights and to explore the treatment outcomes among children diagnosed with MIS-C.

## 5. Conclusions

Receiving antiviral treatment during COVID-19 infection was significantly associated with a reduced incidence of MIS-C in children. Accurate evidence of COVID-19 exposure is crucial for facilitating timely diagnosis and management, thereby enhancing the outcomes for children with MIS-C.

## Figures and Tables

**Table 1:** Demographic characteristics among MIS-C case children and hospitalized control children with COVID-19 infection.

Variable	MIS-C Group (n = 34)	Control Group (n = 68)	*p*-Value
**Gender, *n* (%)**			1
Female	16 (47.1%)	32 (47.1%)	
Male	18 (52.9%)	36 (52.9%)	
**Age at the time of COVID-19 infection (years), median (IQR)**	6.7 (3.1, 11.8)	5.8 (2.9, 10.5)	0.64
**Percentage of weight for height, median (IQR)**	94.5 (84.5, 108.5)	101.1 (89.2, 119.6)	0.23
**Nutrition status, *n* (%)**			0.63
Normal	13 (38.2%)	27 (39.7%)	
Malnutrition	13 (38.2%)	18 (26.5%)	
Overweight	5 (14.7%)	15 (22.1%)	
Obesity	3 (8.8%)	8 (11.8%)	
**Received COVID-19 vaccination, *n* (%)**			0.26
No	25 (73.5%)	47 (69.1%)	
1 dose	4 (11.8%)	16 (23.5%)	
More than 1 doses	5 (14.7%)	5 (7.4%)	
**Duration** **after 1st dose of vaccine and COVID-19 infection (days), median (IQR)**	42 (32, 122)	71 (22, 118)	0.88
**Duration after 2nd dose of vaccine and COVID-19 infection (days), median (IQR)**	175 (132, 217)	91.5 (83, 103)	0.53
**Duration after last dose of vaccine and COVID-19 infection (days), median (IQR)**	42 (32, 59)	86 (29, 114)	0.35
**Duration after 1st dose of vaccine and MIS-C diagnosis (days), median (IQR)**	149 (56, 334)	-	-
**Duration after 2nd dose of vaccine and MIS-C diagnosis (days), median (IQR)**	309 (127, 533)	-	-
**Duration after last dose of vaccine and MIS-C diagnosis (days), median (IQR)**	109 (56, 127)	-	-

**Table 2:** Clinical characteristics during hospitalized from COVID-19 infection the case of group with MIS-C and control group.

Variable	MIS-C Group (n = 18)	Control Group (n = 68)	*p*-Value
**Upper respiratory tract symptoms, *n* (%)**	6 (33.3%)	43 (64.2%)	0.019
**Lower respiratory tract symptoms, *n* (%)**	5 (27.8%)	41 (60.3%)	0.014
**Gastrointestinal tract symptoms, *n* (%)**	2 (11.1%)	14 (20.6%)	0.51
**Fever, *n* (%)**	12 (66.7%)	45 (66.2%)	0.97
**COVID-19 severity, *n* (%)**			0.05
Mild	16 (88.9%)	52 (76.5%)	
Moderate	1 (5.6%)	16 (23.5%)	
Severe	1 (5.6%)	0 (0.0%)	
**COVID-19 complication, *n* (%)**	1 (5.6%)	4 (5.9%)	1
**White blood cell count (cells/mm^3^), median (IQR)**	8300 (7315, 8915)	7750 (5655, 9185)	0.48
**Absolute neutrophil count (cells/mm^3^), median (IQR)**	5746 (4847, 6193)	2905 (2051, 4591)	0.12
**Absolute lymphocyte count (cells/mm^3^), median (IQR)**	996 (882, 1660)	2949 (1907, 4551)	0.041
**Blood platelets (cells/mm^3^), median (IQR)**	145,000 (140,000, 176,000)	287,000 (253,000, 317,500)	0.011
**Received anti-viral therapy during COVID-19 infection, *n* (%)**	8 (44.4%)	53 (77.9%)	0.005

**Table 3:** Univariate and multivariate analyses reflecting factors associated with MIS-C in children.

Variables	Crude OR (95% CI)	Adjusted OR (95% CI)
**Received anti-viral therapy during COVID-19 infection**		
Not received	Reference	Reference
Received	0.13 (0.06–0.30)	0.06 (0.02–0.20)
**Nutritional status**		
Normal	Reference	Reference
Malnutrition	1.37 (0.63–2.96)	0.79 (0.23–2.70)
Overweight	0.74 (0.26–2.07)	0.93 (0.22–3.96)
Obesity	0.81 (0.23–2.85)	0.44 (0.07–2.92)
**Coronavirus 2019 vaccine**		
Not received	Reference	Reference
Received 1 dosed	0.50 (0.17–1.51)	0.28 (0.06–1.27)
Received at least 2 doses	1.53 (0.57–4.12)	1.83 (0.34–9.89)

## Data Availability

The datasets generated and/or analyzed during the current study are not publicly available but are available from the first author (B.P.) upon request.

## Abbreviations

The following abbreviations are used in this manuscript:

MIS-C	Multisystem Inflammatory Syndrome in Children
CDC	Centers for Disease Control and Prevention
SARS-CoV-2	Severe Acute Respiratory Syndrome Coronavirus-2
WHO	World Health Organization
NHS	National Health Service
RCPCH	Royal College of Pediatrics and Child Health
PIM-TS	Pediatric Inflammatory Multisystem Syndrome Temporally Associated with SARS-CoV-2
CRP	C-reactive protein
ESR	Erythrocyte sedimentation rate
LDH	Lactate dehydrogenase
IL-6	Interleukin-6
IQR	Interquartile ranges
95% CI	95% confidence interval
